# Supplementary material for: Antioxidant Activity of an Aqueous Leaf Extract from Uncaria tomentosa and Its Major Alkaloids Mitraphylline and Isomitraphylline in Caenorhabditis elegans
Source: Molecules. 2019 Sep 10;24(18):3299. doi: 10.3390/molecules24183299 (PMC6766911; doi:10.3390/molecules24183299)
Supplement: Supplementary file 1 [file molecules-24-03299-s001.pdf]

molecules

Supplementary Information

Identification of isorhynchophylline by NMR

The oxindole alkaloids rhynchophylline and isorhynchophylline belong to *normal serie:* C-3 (α), C-15 (α) and C-20 (β). The ^{13}C-NMR spectrum showed that the chemical displacement of C-3 of isorhynchophylline (δ 72.0) differ from its isomer rhynchophylline (δ 75.3) due the effect of carboxyl group presence into oxindole moiety (Table S1; Figure S4). The ^{1}H NMR spectra confirmed the chemical structure from isorhynchophylline as well (Figure S1-S3).

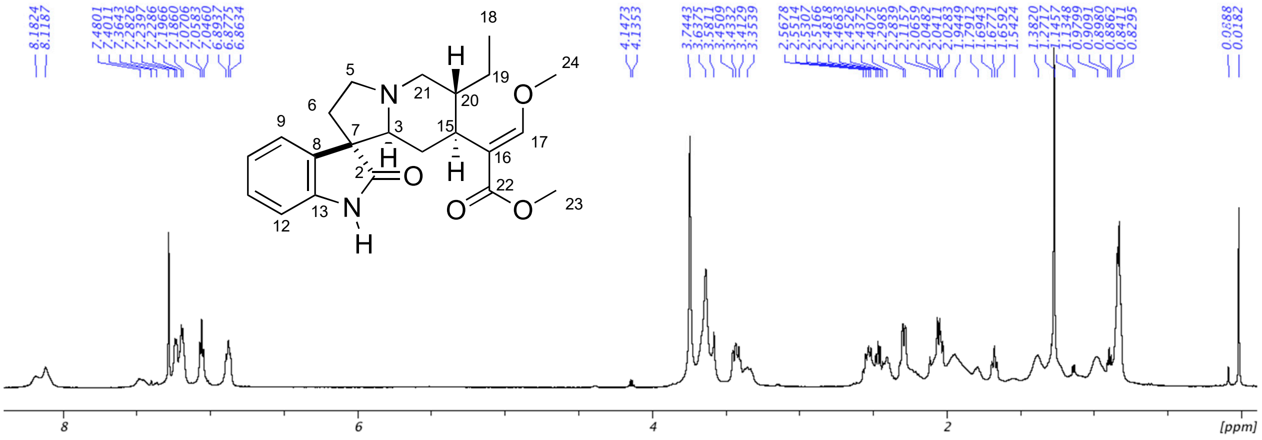

Figure S1. ^{1}H-NMR spectrum of isorhynchophylline (600 MHz, DMSO-d_6).

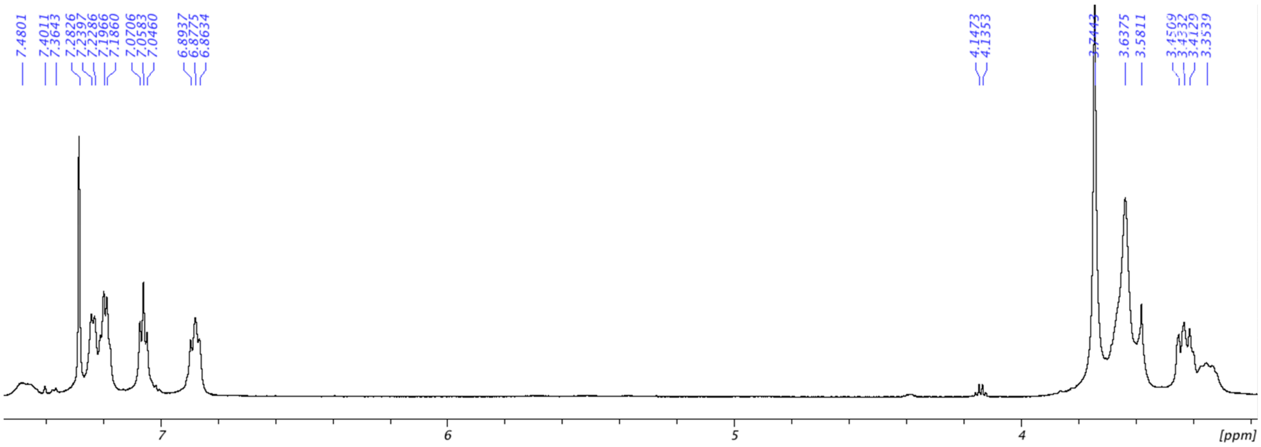

Figure S2. ^{1}H-NMR spectrum (expanded view) of isorhynchophylline (600 MHz, DMSO-d_6).

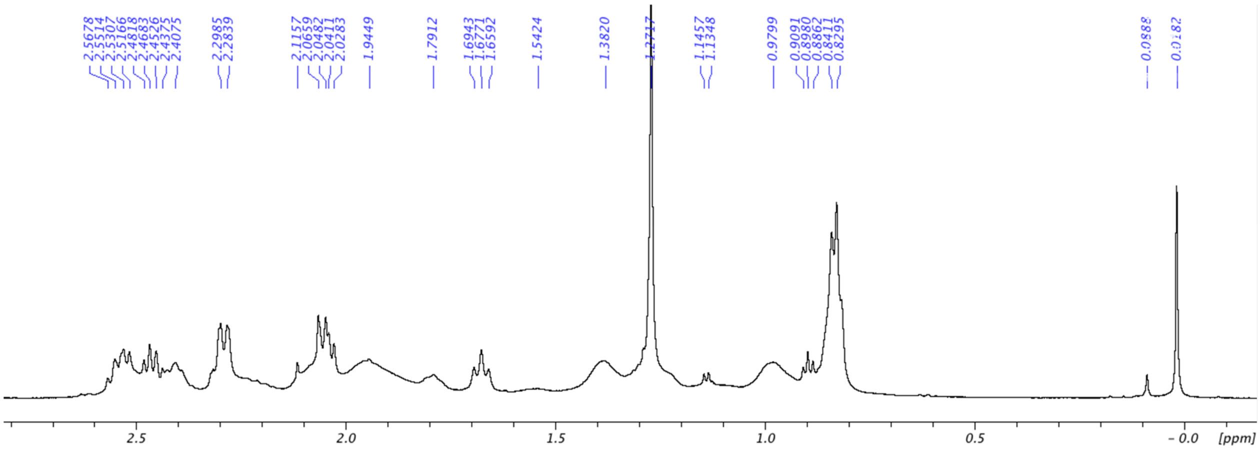

Figure S3.^{1}H-NMR spectrum (expanded view) of isorhynchophylline (600 MHz, DMSO-d_6).

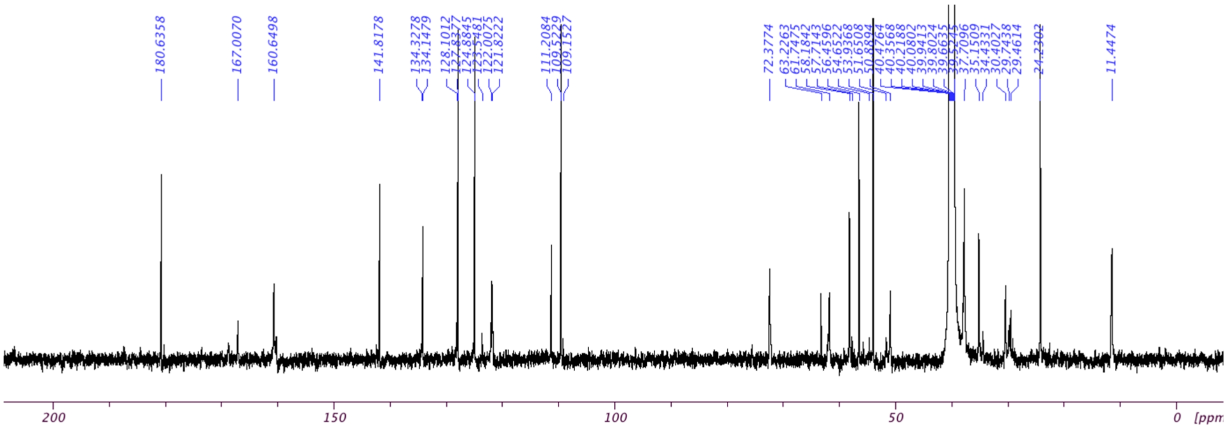

Figure S4. ^{13}C NMR (150 MHz, DMSO-d_6) spectrum of isorhynchophylline.

Table S1. ^{13}C-NMR spectral data of isorhynchophylline isolated from *U. tomentosa* (150 MHz, DMSO-d_6).

C	*isorhynchophylline*	*isorhynchophylline**	*rhynchophylline**
**2**	180.6	181.9	181.6
**3**	72.3	72.3	75.3
**5**	53.9	54.2	55.0
**6**	35.1	35.6	34.8
**7**	56.4	56.8	55.1
**8**	134.3	134.0	133.9
**9**	124.8	125.2	123.1
**10**	121.8	122.3	122.4
**11**	128.1	127.4	127.7
**12**	109.5	109.2	109.4
**13**	141.8	140.1	141.1
**14**	30.4	30.3	29.0
**15**	37.7	37.6	37.9
**16**	111.2	112.4	111.9
**17**	160.6	159.5	159.7
**18**	11.4	11.2	11.3
**19**	24.2	24.2	24.2
**20**	37.7	38.2	39.7
**21**	57.7	58.2	58.2
**22**	167.0	168.0	169.0
**23-OCH$_3$**	50.8	50.6	51.1
**24- OCH$_3$**	61.7	61.2	61.4
*** literature**			

*Sakakibara, I., Takahashi, H., Terabayashi, S., Yuzurihara, M., Kubo, M., Ishigel, A., Higuchi, M., Komatsu, Y., Okada, M., Maruno, M., Biqiang, C., Jiang, H. X. Effect of oxindole alkaloids from the hooks of *Uncaria macrophylla* on thiopental-induced hypnosis. *Phytomedicine*, 52, 83-86, 1998.
